# Transgene-Free Genome Editing in Tomato and Potato Plants Using *Agrobacterium*-Mediated Delivery of a CRISPR/Cas9 Cytidine Base Editor

**DOI:** 10.3390/ijms20020402

**Published:** 2019-01-18

**Authors:** Florian Veillet, Laura Perrot, Laura Chauvin, Marie-Paule Kermarrec, Anouchka Guyon-Debast, Jean-Eric Chauvin, Fabien Nogué, Marianne Mazier

**Affiliations:** 1 INRA, Agrocampus Ouest, Université Rennes 1, UMR 1349 IGEPP, Domaine de Kéraïber, 29260 Ploudaniel, France; florian.veillet@inra.fr (F.V.); laura.chauvin@inra.fr (L.C.); marie-paule.kermarrec@inra.fr (M.-P.K.); jean-eric.chauvin@inra.fr (J.-E.C.); 2 INRA PACA, UR 1052, GAFL unit (Génétique et Amélioration des Fruits et Légumes), 84143 Montfavet, France; laura.perrot@inra.fr; 3 Institut Jean-Pierre Bourgin, INRA, AgroParisTech, CNRS, Université Paris-Saclay, 78000 Versailles, France; anouchka.guyon-debast@inra.fr (A.G.-D.); fabien.nogue@inra.fr (F.N.)

**Keywords:** CRISPR/Cas9, cytidine base editor, *Agrobacterium*, *ALS*, transgene-free, potato, tomato

## Abstract

Genome editing tools have rapidly been adopted by plant scientists for gene function discovery and crop improvement. The current technical challenge is to efficiently induce precise and predictable targeted point mutations valuable for crop breeding purposes. Cytidine base editors (CBEs) are CRISPR/Cas9 derived tools recently developed to direct a C-to-T base conversion. Stable genomic integration of CRISPR/Cas9 components through *Agrobacterium*-mediated transformation is the most widely used approach in dicotyledonous plants. However, elimination of foreign DNA may be difficult to achieve, especially in vegetatively propagated plants. In this study, we targeted the *acetolactate synthase* (*ALS*) gene in tomato and potato by a CBE using *Agrobacterium*-mediated transformation. We successfully and efficiently edited the targeted cytidine bases, leading to chlorsulfuron-resistant plants with precise base edition efficiency up to 71% in tomato. More importantly, we produced 12.9% and 10% edited but transgene-free plants in the first generation in tomato and potato, respectively. Such an approach is expected to decrease deleterious effects due to the random integration of transgene(s) into the host genome. Our successful approach opens up new perspectives for genome engineering by the co-edition of the *ALS* with other gene(s), leading to transgene-free plants harboring new traits of interest.

## 1. Introduction

Genome editing tools, mostly based on the CRISPR/Cas9 system, have rapidly emerged in plants for gene function analysis and to improve agronomical traits. This technology relies on the creation of DNA double-strand breaks (DSBs) at predefined targeted sites in the plant genome. DSBs can be repaired by an error-prone non-homologous end-joining (NHEJ) mechanism that may result in small insertions or deletions (indels) in the targeted sequence, leading to gene knock-out, an essential tool for gene function analysis. A refinement of genome editing consists of the precise and predictable point mutations that are even more promising to decipher natural genomic variations and for crop breeding purposes. This could be achieved via homologous recombination (HR), but this repair mechanism suffers from low efficiency in plants and the delivery of donor DNA templates in plant cells, needed to promote the process, is still challenging [1]. More recently, base editors have been established based on either fusing an adenine or a cytidine deaminase to a Cas9 nickase (nCas9), leading to an A-to-G or a C-to-T substitution, respectively, without the introduction of a DSB. These base editing systems rely on the association of a single-guide RNA molecule (sgRNA) with the nCas9/deaminase fusion, driving the complex to the target locus and enabling deamination on the non-complementary strand [2]. To date, base editors have been successfully used in a number of crops, including rice, tomato, potato, wheat, maize, rape and watermelon [3,4,5,6,7,8,9,10,11].

For the delivery of CRISPR/Cas9 components into dicotyledonous plant cells, stable genomic integration of expression units through *Agrobacterium*-mediated transformation is the most widely used approach. When working on sexually propagated plants like tomato, the transfer DNA (T-DNA) can be eliminated thanks to Mendelian segregation in the subsequent generations, resulting in edited but transgene-free plants. However, this strategy cannot be applicable to vegetatively propagated and/or highly heterozygous plants like potato, as sexual reproduction would lead to the loss of desirable traits. To circumvent these limitations, Chen et al. [12] have recently developed a labor-intensive method based on the delivery of CRISPR/Cas9 reagents by *Agrobacterium*-mediated transformation followed by a high-throughput screening protocol in the tetraploid tobacco, leading to transgene-free mutants without selective pressure. Although some alternative delivery methods such as particle bombardment or protoplast transfection using plasmid DNA or ribonucleoprotein (RNP) complexes have been demonstrated to result in non-transgenic mutants, these approaches are often highly sensitive and limited to some species due to bottlenecks in the regeneration process. Regeneration from protoplasts has been recently shown to result in a substantial genome instability in the tetraploid potato, affecting the prospects for protoplast utilization in genome editing for this species [13]. Whether this syndrome may be reduced using regeneration from *Agrobacterium*-transformed explants is a question of upmost importance. Furthermore, if protoplast transfection could be considered “foreign-DNA-free”, the need for a large amount of plasmid DNA during protoplast transfection may result in the insertion of degraded DNA fragments into the target site or elsewhere into the genome [14,15,16,17].

The *acetolactate synthase* (*ALS*) gene encodes the enzyme that catalyses the initial step of the biosynthetic pathway for branched-chain amino acids. As many studies have shown that point mutations in this gene can confer dominant resistance to ALS-inhibitors, the targeting of the *ALS* gene constitutes a tool of choice for the selection of edited plants. The mutation of the Proline-197 residue (amino acid number standardized to the *Arabidopsis* sequence, corresponding to Proline-186 in tomato and potato *ALS1*) is one of the most commonly reported mutations conferring chlorsulfuron resistance [18]. According to the online database of sulfonylurea-resistant weed populations (http://www.weedscience.com/), chlorsulfuron resistance can be obtained by at least 11 different amino acids substitutions at Pro-197. In order to test and potentially improve the production of transgene-free edited plants that can be selected directly in vitro, we targeted the *ALS* locus in tomato and potato through *Agrobacterium*-mediated transformation using Target-AID (target-activation-induced cytidine deaminase), a cytidine base editor (CBE) [5].

## 2. Results and Discussion

### 2.1. Highly Efficient Production of Base Edited and Transgene-Free Tomatoes

**Figure 1:**
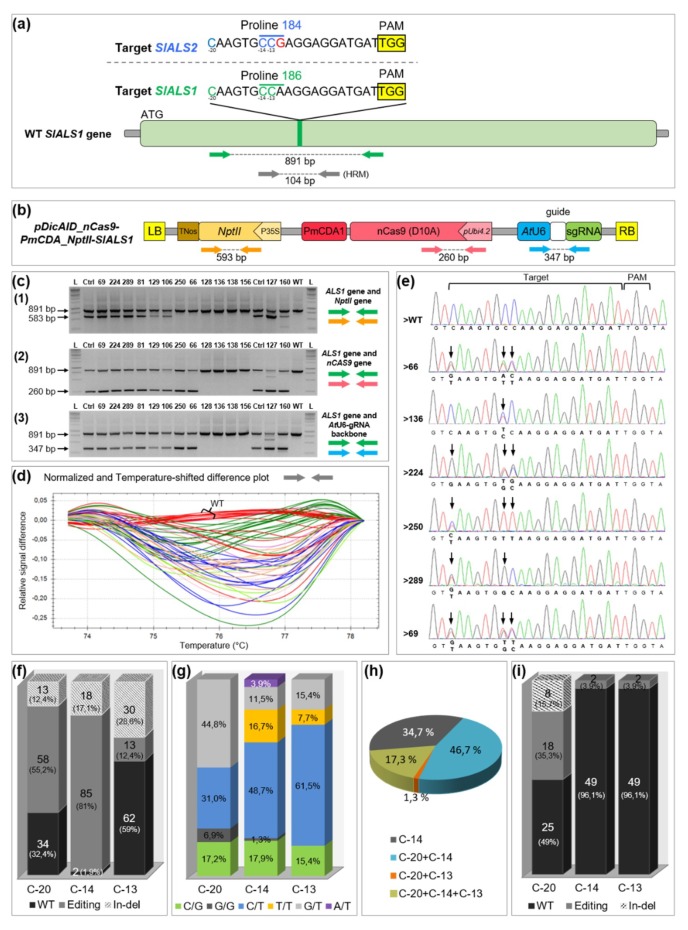
Targeted modifications in tomato *StALS1* gene. (**a**) *SlALS1* and *SlALS2* target region. The *SlALS1* Pro186 codon is highlighted in green and the *SlALS2* Pro184 codon in blue. The single mismatch in *SlALS2* locus is written in red. The three targeted cytidines (C_−20_, C_−14_ and C_−13_) are written in green and in blue in the *SlALS1* and *StALS2* sequences, respectively. The protospacer adjacent motif (PAM) site is highlighted in yellow. Green and grey arrows indicate the relative positions of the PCR primers; (**b**) T-DNA physical map of the cytidine base editor (CBE) binary vector. Colored arrows indicate the relative positions of the PCR primers; (**c**) multiplex PCR analyses of 14 independent chlorsulfuron resistant plants, the wild-type (WT) and the positives controls (T+). L: molecular marker; (**d**) high resolution melting (HRM) assay using primers (grey arrows) flanking the targeted *SlALS1* region. The color-label represents groups of plants (40 plantlets) that harbor similar melting curve shapes. The wild-type curves are colored in red. (**e**) chromatograms of the targeted region of the WT and of six independent chlorsulfuron resistant plants. The arrows indicate modifications; (**f**) histograms indicating the number of unedited plants (black), edited plants (grey) and plants with indels (in-del, striped), independently for C_−20_, C_−14_ or C_−13_ (not for the entire sequence), found on a total of 105 mutated plants in the targeted *SlALS1* region; (**g**) percentage of each type of nucleotide changes found on cytidines C_−20_, C_−14_ or C_−13_. The total number of edited plants used in this analysis was 58 for C_−20_, 78 for C_−14_, 13 for C_−13_; (**h**) percentage of single, double and triple editing events in 75 base edited plants; (**i**) histogram indicating the number of unedited plants (in black), edited plants (in grey), and plants with indels (in-del, striped) in the *SlALS2* locus, independently for C_−20_, C_−14_ or C_−13_ (not for the entire sequence), found on a total of 51 mutated plants on the *SlALS1* locus.

The base editing strategy was firstly deployed on the diploid tomato. Because *Agrobacterium* is sometimes used for transient expression of transcriptional units located on the T-DNA [19], we developed a specific selection protocol in order to exploit this potentiality to obtain T-DNA-free events by transiently expressing the CBE. Based on the *Solanum lycopersicum* reference genome, we designed one sgRNA targeting the *SlALS1* gene (Solyc03g044330), ensuring that nucleotides encoding the Pro186 (CCA) were located in the edition window of the CBE (Figure 1a, Figures S1 and S2). Because of the very high similarity between *SlALS1* and *SlALS2* genes, a single mismatch at the limit of the seed region of the sgRNA, at position -12 counting from the protospacer adjacent motif (PAM), was present with the *SlALS2* gene (Solyc07g061940). The guide was cloned into the CBE binary vector [5], and *Agrobacterium*-mediated transformation was performed (Figure 1b). After one or two weeks of kanamycin selection pressure covering the transient expression period of *Agrobacterium*, plant tissues were transferred to a selective medium containing 40 ng mL^−1^ chlorsulfuron, so that only edited cells could grow and regenerate plantlets.

Seventy-six percent of the treated cotyledons (289/378) were able to produce at least one plantlet with a strong resistance to chlorsulfuron. The first 232 plantlets that regenerated and rooted on chlorsulfuron media, all corresponding to independent transformation events, were first checked by polymerase chain reaction (PCR) for T-DNA integration (Figure 1c). Thirty plantlets out of the 232 chlorsulfuron resistant plants were found T-DNA-free (Figure S3). A hundred and five plants, out of the 232 chlorsulfuron resistant plants and including the 30 “T-DNA free” plants, were analysed for edition efficiency, firstly by High Resolution Melting analysis (HRM). All these plants displayed a mutated melting-curve shape compared to the wild-type at the *SlALS1* targeted locus (Figure 1d). To characterize the base editing outcomes, the target site was then sequenced in these plants.

Counting starting from the PAM site, three cytidines are present in the edition window of the sgRNA sequence: C_−20_, C_−14_ and C_−13_, the last two corresponding to the Pro186 CCA codon. Ninety-nine percent (104/105) of the analysed sequences displayed mutation(s) at the *SlALS1* locus. One plant, in which no C was found modified, died a few weeks after sequencing and was probably a chlorsulfuron selection escape. Up to 28.5% of the sequences showed indels in the target site, mostly originating from the edition window, where cytidine deamination is supposed to occur (Figure 1e,f and Figure S4). The formation of such a rate of undesired indels is in accordance with previously published results in tomato with the same base editor [5], and prevented a proper analysis of the editing outcomes at the C positions affected by indels. Seventy-five out of 105 plants (71.4%) were cleanly base edited at the *SlALS1* Pro186 (CCA) codon (by extrapolating, ≈ 54% of the agroinoculated cotyledon explants). From these, 98.7% (74/75) were edited at position C_−14_. Any substitution (C-to-T, C-to-A or C-to-G) at this position is sufficient to change Pro186 amino acid to Ser, Ala or Thr residues that have been shown to confer chlorsulfuron resistance in tobacco (Figure 1e and Figure S5) [20]. More than 80% of edited C_−14_ were C-to-T changes, 16.7% being homozygous (Figure 1g; plant >250 Figure 1e). As expected, no C_−20_-to-T_−20_ homozygous modification was found as it would lead to amino acid change toward a stop codon, likely preventing plant regeneration. As a whole, most of the analysed plants were modified at several C positions (Figure 1h; plants >66, >224, >250, >69 Figure 1e). Only 34.7% of the analysed sequences showed a single C substitution, and exclusively at the C_−14_ targeted position.

In tobacco, natural mutations conferring chlorsulfuron resistance and centered on the Pro codon, equivalent to the tomato Pro186 and Pro184 ones, have been found in the *ALS1*, but also in the *ALS2* gene [21]. It has been shown that off-targeting in potato *ALS2* gene can occur, even with one mismatch within the seed region of the sgRNA [22]. Because of the presence of a mismatch at the limit of the seed region (N_−12_) between the sgRNA and the *SlALS2* gene, we sequenced 51 plants at this locus, including most of the T-DNA free genotypes (Figure S6). Nineteen sequences were found with base editing events (37%) and eight with indels (16%) (Figure 1i). Most of the base editing events (18/19) were observed at position C_−20_ while two base conversion events were unexpectedly found at position C_−24_, upstream of the sgRNA sequence (Figures S6 and S7). The lower but nevertheless substantial amount of edition events at *SlALS2* locus as compared to *SlALS1* target site demonstrates that the off-target potential should be carefully estimated while designing target sequences [23]. Interestingly, among the 25 analysed T-DNA-free *SlALS1* mutants, only four (16%) were edited at the *SlALS2* locus (Figure S6). In contrast, the number of plants edited at the *SlALS2* locus in the transgenic plants, 23 (88%) out of the 26 analysed, is significantly higher (Fisher exact test *p* = 0.003). These results suggest that limiting the expression of the CRISPR components to a few days strongly reduce the risk of off-target (5-fold decrease in our case) in tomato.

In summary, a very high base editing efficiency was obtained with the CBE strategy in tomato, allowing the recovery of T-DNA free edited plants at a reasonable rate (12.9% of the analysed chlorsulfuron-resistant plants), in the same range as a recently developed strategy in tobacco [12].

### 2.2. Production of Transgene-Free Base Edited Plants in the Tetraploid Potato

**Figure 2:**
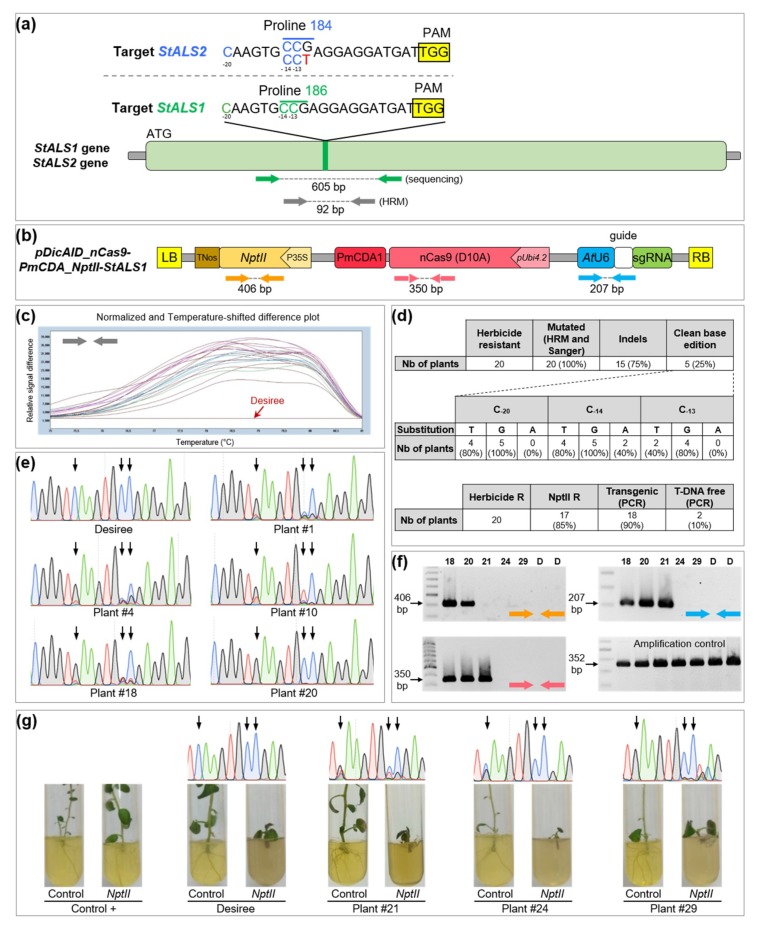
Targeted modifications in potato *StALS* gene. (**a**) target region in the *StALS1* and *StALS2* genes of the cultivar Desiree. The *StALS1* Pro186 codon is highlighted in green and the *StALS2* Pro184 codon in blue. The single mismatch in the *StALS2* locus is highlighted in red. Targeted C_−20_, C_−14_ and C_−13_ are represented in green and blue for *StALS1* and *StALS*2, respectively. PAM site is highlighted in yellow. Green and grey arrows indicate the relative positions of the PCR primers; (**b**) T-DNA physical map of the CBE binary vector. Colored arrows indicate the relative positions of the PCR primers; (**c**) HRM analysis using primers targeting both *StALS1* and *StALS2* loci. Plants (20) are color-labeled according to their melting curve shape. The wild-type curve is colored in red; (**d**) summary of mutation efficiency and outcomes for 20 regenerated plants. The number of edited and transgenic or T-DNA-free plants is indicated; (**e**) Sanger chromatograms of the targeted region (*StALS1* and *StALS2*) of five plants that do not harbor indels. The wild-type sequence is also provided. The black arrows indicate the localization of targeted cytidines; (**f**) PCR analyses of five chlorsulfuron resistant plants and the wild-type using primers matching the T-DNA (localization is indicated by the colored arrows); (**g**) Sanger chromatograms and rooting test of the three plants that did not amplify the *NptII* fragment. The wild-type and a positive control are also included. The black arrows indicate the localization of the targeted cytidines.

We have shown that our base editing strategy was efficient in the diploid tomato. Next, we aimed to apply the same approach in another *Solanaceous* species: the tetraploid, highly heterozygous and vegetatively propagated potato. The production of edited plants without stable integration of foreign DNA in such a species is an even more promising perspective, as transgene segregation through selfing cannot be performed without changing cultivar characteristics. Based on the potato reference genome, we simultaneously sequenced a portion of *StALS1* (PGSC0003DMG400034102) and *StALS2* (PGSC0003DMG400007078) homologs (96% protein identity; Figure S8) in the potato cultivar Desiree, using one couple of primers predicted to match the two genes (Figure 2a). Then, we designed one sgRNA targeting the *StALS1* gene and covering the Pro-186 codon, while one mismatch was present at the limit of the seed region with some alleles of the *StALS2* locus (Figure 2a). The guide was cloned into the CBE (Figure 2b) and an *Agrobacterium*-mediated transformation was performed in potato explants. After two weeks of kanamycin selection pressure, plant tissues were transferred to a selective medium containing 40 ng mL^−1^ chlorsulfuron.

Twenty plants were regenerated and confirmed to be chlorsulfuron-resistant. Then, the HRM analysis showed that all the chlorsulfuron-resistant plants were mutated at the targeted locus (Figure 2c,d), showing that our editing selection strategy was highly efficient in potato. By contrast, base-editing efficiency in watermelon was 23% in the T0 generation, highlighting the advantage of selecting primary transformants on medium-containing an ALS-inhibitor [7]. All these plants were subjected to direct Sanger sequencing using primers matching both *StALS1* and *StALS2* genes. Sanger results confirmed that all plants harbored mutation(s) in the target sequence (Figure 2d). As previously observed in tomato, we noticed that a significant part of the potato plants (15 out of 20 mutated plants, 75%) showed indels in the target site (Figure 2d, Figures S9 and S10), which likely originate from uracil excision and downstream repair systems [24]. This substantial rate of indels is not surprising due to the number of targeted cytidines in each of the eight *StALS* alleles. Addition of an uracil DNA glycosylase inhibitor protein (UGI) to the deaminase function may prevent the formation of such undesired products [24], as observed in watermelon where the same *ALS* site was targeted using another cytidine base editor that harbored an UGI [7]. For a rigorous analysis of base editing, we decided to focus our analysis on the five plants without indels. Base conversion was mainly C-to-G and C-to-T, while C-to-A was much less frequent (Figure 2d,e). Based on a visual peak intensity analysis, we noticed that base conversion at C_−20_ and C_−14_ occurred more frequently than at C_−13_ (Figure 2e), as previously observed for tomato. For plant #10, chromatogram analysis showed that the C_−20_ was totally converted (Figure 2e), suggesting that the CBE is sufficiently efficient to mediate base conversion on the eight alleles.

To discriminate transgenic from non-transgenic edited plants, we performed PCR on the 20 *StALS* mutants using primers covering three regions in the T-DNA (Figure 2f). Two plants (10%) were considered to be T-DNA-free as they lacked all three PCR fragments. One plant harbored a truncated integration of the transgene, with a missing left border that contains the *nptII* cassette (Figure 2f). The growth of these three plants was strongly impaired under kanamycin treatment compared to transgenic plants, confirming our molecular analysis (Figure 2g).

## 3. Materials and Methods

### 3.1. Vector Cloning

Guide sequences targeting the *ALS* gene for tomato and potato were cloned into the pDicAID_nCas9-PmCDA_NptII_DELLA [5]. First, a 310 bp fragment containing the 3′ end of the AtU6-26 promoter, the 20 bp guide and the sgRNA scaffold were synthesized (Genscript, Piscataway, NJ, USA, for potato; IDT, Skokie, IL, USA, for tomato). *BstXI* and *SpeI* restriction sites were added at the 5′ and the 3′ end of the fragment, respectively. Synthesized fragment was subcloned into the pDONR207 using BP reaction (Thermo Fischer Scientific, Waltham, MA, USA). Then, the resulting pDONR207 and the binary plasmid were digested by the FastDigest restriction enzymes *BstXI* and *SpeI* (Thermo Fischer Scientific, Waltham, MA, USA). The synthesized fragment was ligated into the digested binary vector using T4 DNA ligase (New England Biolabs, Ipswich, MA, USA). Reaction mixture was transformed into One Shot™ TOP10 Chemically Competent *E. coli* (Thermo Fisher Scientific, USA) and bacteria were grown overnight at 37 °C on LB plates containing 100 µg mL^−1^ spectinomycin. Plasmids were Sanger sequenced (Genoscreen, Lille, France) and transferred into *Agrobacterium tumefaciens* by heat shock.

### 3.2. Agrobacterium-Mediated Transformation

Tomato plants were cultured in sterile conditions in a growth chamber with controlled temperatures of 22 °C/18 °C under a 16 h/8 h (day/night) photoperiod. *Agrobacterium*-mediated transformation using the C58 pGV2260 strain containing the CBE binary vector with *SlALS1* sgRNA was performed on cotyledon segments from 8–12 day-old seedlings of the WVA106 tomato cultivar, as previously described [25]. After selection on kanamycin (100 mg L^−1^) for one or two weeks, the cotyledon pieces were transferred to fresh selective medium containing 40 ng mL^−1^ chlorsulfuron every two weeks.

The tetraploid potato cultivar Desiree (ZPC, the Netherlands) was in vitro propagated in 1X Murashige and Skoog (MS) medium (pH 5.8) including vitamins (Duchefa, the Netherlands), 0.4 mg L^−1^ thiamine hydrochloride (Sigma-Aldrich, Saint-Louis, MO, USA), 2.5% sucrose and 0.8% agar powder (VWR, Radnor, PA, USA). Plants were cultivated in a controlled environmental chamber at 19 °C under a 16 h light/8 h dark photoperiod. Stem and petiole tissues were cut from the top of 3 to 5-week-old plants, and placed overnight in the growth chamber on 1X Murashige and Skoog (MS) medium (pH 5.8) including vitamins (Duchefa, Haarlem, the Netherlands), 2.5% sucrose, 0.4 mg L^−1^ thiamine hydrochloride (Sigma-Aldrich, USA), 1 mg L^−1^ indole-3-acetic acid (Sigma-Aldrich, USA), 1 mg L^−1^ zeatin-riboside (Sigma-Aldrich, USA), 1 mg L^−1^ gibberellin A3 (Sigma-Aldrich, USA) and 0.7% agar powder (VWR, Radnor, PA, USA). *A. tumefaciens* C58pMP90 strain containing the CBE binary vector with the *StALS1* sgRNA was grown overnight and the bacterial DO was set to ≈0.2. Potato tissues were co-cultured with *Agrobacterium* for 48 h at 25 °C in the dark, washed with sterile water and placed onto the culture medium described above, supplemented with 250 µg mL^−1^ cefotaxime, 100 µg mL^−1^ timentin® and 50 µg mL^−1^ kanamycin. After two weeks, explants were transferred onto a fresh culture medium with 40 ng mL^−1^ chlorsulfuron and reduced indole-3-acetic acid (0.1 mg L^−1^), and subcultured every three weeks.

### 3.3. Genotyping Analysis

Detection of the stable integration of the T-DNA was detected by PCR analysis from genomic DNA, using the GoTaq^®^ G2 Flexi DNA Polymerase (Promega, Madison, WI, USA).

The High Resolution Melting-curve analysis was mostly performed as described in Veillet et al. [26]. Briefly, PCR amplification was carried out from genomic DNA using small amplification products (≈100 bp). For tomato, the experiment was carried out using the Precision Melt Supermix (BioRad, Hercules, CA, USA) with the CFX96™ Real-Time PCR Detection System (BioRad, Hercules, CA, USA), according to the manufacturer’s instructions. Melting-curves analysis was performed using the Precision Melt Analysis software (BioRad, Hercules, CA, USA). Sequence information was obtained by amplifying the target locus with GoTaq^®^ G2 Flexi DNA Polymerase (Promega, Madison, WI, USA) and PCR products were purified and Sanger sequenced (Genoscreen, Lille, France). For potato, the analysis was performed using the High Resolution Melting Master (Roche, Mannheim, Germany) with the LightCycler^®^ 480 II system (Roche, Mannheim, Germany), according to the manufacturer’s instructions. The analysis of results was done with the LightCycler® 480 Gene Scanning Software (Roche, Mannheim, Germany). For mutation detection, the spiking of all samples with 10–20% of wild type DNA was performed. For sequence information, target locus was amplified using Invitrogen Platinum SuperFi DNA Polymerase (Thermo Fischer Scientific, Waltham, MA, USA) and PCR products were purified and Sanger sequenced (Genoscreen, Lille, France).

Primers used in this study are listed in Tables S1 and S2.

## 4. Conclusions

In summary, we have established a straightforward, efficient and cost-effective strategy for the production of edited and transgene-free plants in tomato and potato, using *Agrobacterium*-mediated transient expression of a CBE. This system can be transferred to the other numerous species that are compatible with *Agrobacterium* transformation, expanding the scope of the CRISPR toolbox for generating foreign-DNA-free plants, especially in vegetatively propagated species and trees [27]. The use of another base editing tool, like the adenine base editor that displays much more cleanly base edition than cytidine base editors [8,9,10], should also be possible by targeting A-rich site(s) of the *ALS* gene that can confer resistance to ALS-inhibitors. As multiplex base editing has been reported in tomato and rice [5,9,28], the co-base-editing of the *ALS* gene with another gene of interest should allow the selection of base editing events lacking the undesired insertion of foreign DNA, and thus constitutes a promising prospect for using this tool, especially for vegetatively propagated species. Such a strategy will reduce the potentially deleterious effects of the random integration of the T-DNA into the host genome and will get rid of the constitutive expression of the base editor, thus limiting the risk for off-targets [23].

## Supplementary Materials

Supplementary materials can be found at http://www.mdpi.com/1422-0067/20/2/402/s1.

## Abbreviations

ALS	Acetolactate synthase
CBE	Cytidine base editors
DSBs	DNA double-strand breaks
HR	Homologous recombination
HRM	High resolution melting
NHEJ	Non-homologous end-joining
PAM	Protospacer adjacent motif
PCR	Polymerase chain reaction
RNP	Ribonucleoprotein
T-DNA	Transfer DNA
Target-AID	Target-activation-induced cytidine deaminase
UGI	Uracil DNA glycosylase inhibitor protein
