# Supplementary material for: Transgene-Free Genome Editing in Tomato and Potato Plants Using Agrobacterium-Mediated Delivery of a CRISPR/Cas9 Cytidine Base Editor
Source: Int J Mol Sci. 2019 Jan 18;20(2):402. doi: 10.3390/ijms20020402 (PMC6358797; doi:10.3390/ijms20020402)
Supplement: Supplementary file 1 [file ijms-20-00402-s001.pdf]

Supporting information

>Solyc03g044330 *SlALS1*

ATGGCGGCTGCTGCCTCACCATCTCCATGTTTCTCCAAAACCCTACCTCCATCTTCCTCCAAATCTTCCACCATTCTACCTAG
ATCTACCTTCTCTTTCCACAATCACCCACAAAAAGCCTCACCCCTTCATCTCATCCACGCTCAACATAATCGTCGTGGTTTTG
CCGTTGCCAATGTCGTCATATCCACTACCACCCATAACGACGTTTCTGAACCTGAAACATTCGTTTCCCGTTTCGCCCCTGAC
GAACCCAGAAAGGGTTGTGATGTTCTTGTGGAGGCACTTGAAAGGGAAGGTGTTACGGATGTATTTGCATACCCAGGAGGTGC
TTCTATGGAGATTCATCAAGCTTTGACACGTTCGAATATTATTCGTAATGTGCTACCACGTCATGAGCAAGGTGGTGTGTTTG
CTGCAGAGGGTTACGCACGGGCTACTGGGTTCCCTGGTGTTTGCATTGCTACCTCTGGTCCCGGAGCTACAAATCTTGTTAGT
GGTCTTGCGGATGCTTTGTTAGATAGTATTCCGATTGTTGCTATTACAGGT**CAAGTGCCAAGGAGGATGAT**TGGTACTGATGC
GTTCCAGGAAACGCCTATTGTTGAGGTAACGAGATCTATTACGAAGCATAATTATCTTGTTATGGATGTAGAAGATATTCCTA
GGGTTGTTCGTGAAGCATTTTTTCTTGCGAAATCGGGACGGCCTGGCCCAGTTTTGATTGATGTACCTAAGGATATTCAGCAA
CAATTGGTGATACCTAATTGGGATCAGCCAATGAGGTTGCCTGGTTACATGTCTAGGTTACCTAAATTGCCTAATGAAATGCT
TTTGGAACAAATTGTTAGGCTGATTTCCGAGTCGAAGAAGCCTGTTTTGTATGTGGGTGGTGGGTGTTCGCAATCAAGTGAGG
AGCTGAGACGATTTGTGGAGCTTACAGGTATTCCTGTAGCGAGTACTTTGATGGGTCTTGGAGCTTTTCCAACTGGGGATGAG
CTTTCACTTCAAATGTTGGGTATGCATGGAACTGTGTATGCTAATTATGCTGTGGATAGTAGTGATTTGTTGCTTGCATTTGG
GGTGAGGTTTGATGATCGAGTTACTGGTAAATTGGAAGCTTTTGCTAGTCGAGCGAAAATTGTCCACATTGATATTGATTCGG
CAGAGATTGGAAAAAACAAGCAACCTCATGTTTCCATTTGTGCAGATATCAAGTTGGCATTACAGGGTTTGAATTCCATATTG
GAGGGTAAAGAAGGTAAGATGAAGTTAGATTTTTCTGCCTGGAGGCAGGAGTTAACGGAGCAGAAGATGAAGTACCCACTGAA
TTTTAAGACTTTTGGTGATGCCATCCCTCCACAATATGCTATTCAGGTTCTTGATGAGTTAACTAACGGAAATGCCATTATTA
GTACTGGTGTGGGGCAACACCAGATGTGGGCTGCCCAATACTATAAGTACAAAAAGCCACGCCAATGGTTGACATCTGGTGGA
TTAGGAGCAATGGGATTTGGTTTGCCTGCTGCTATAGGTGCGGCTGTTGGGAGACCGGGTGAGATTGTGGTTGACATTGACGG
TGATGGGAGTTTTATCATGAATGTGCAAGAGTTAGCAACAATTAAGGTGGAGAATCTCCCAGTTAAGATTATGTTGCTGAATA
ATCAACACTTGGGAATGGTGGTTCAATGGGAGGATCGATTCTATAAAGCTAACAGAGCACACACTTACTTGGGTGACCCTTCT
AACGAGGAAGAGATCTTCCCTAATATGTTGAAATTTGCAGAGGCTTGTGGCGTACCTGCTGCAAGAGTGTCACACAGGGATGA
TCTTAGAGCTGCCATTCAAAAGATGTTAGACACTCCTGGGCCATACTTGTTGGATGTGATTGTACCTCATCAGGAGCACGTTC
TACCTATGATTCCCAGCGGTGGTGCTTTCAAAGATGTGATCACGGAGGGCGATGGGAGATGTTCCTAT**TGA**

Figure S1. *SlALS1* wild-type gene sequence

The start codon is represented with red letters and the stop codon is represented with bold letters. The target sequence is highlighted in blue. The three targeting cytidines are highlighted in red. The PAM site is highlighted in green.

>Solyc03g044330 protein SlALS1

MAAAASPSPCFSKTLPPSSSKSSTILPRSTFSFHNHPQKASPLHLIHAQHNRRGFAVANVVISTTTHNDVSEPETFVSRFAPD
EPRKGCDVLVEALEREGVTDVFAYPGGASMEIHQALTRSNIIRNVLPRHEQGGVFAAEGYARATGFPGVCIATSGPGATNLVS
GLADALLDSIPIVAITGQVPRRMIGTDAFQETPIVEVTRSITKHNYLVMDVEDIPRVVREAFFLAKSGRPGPVLIDVPKDIQQ
QLVIPNWDQPMRLPGYMSRLPKLPNEMLLEQIVRLISESKKPVLYVGGGCSQSSEELRRFVELTGIPVASTLMGLGAFPTGDE
LSLQMLGMHGTVYANYAVDSSDLLLAFGVRFDDRVTGKLEAFASRAKIVHIDIDSAEIGKNKQPHVSICADIKLALQGLNSIL
EGKEGKMKLDFSAWRQELTEQKMKYPLNFKTFGDAIPPQYAIQVLDELTNGNAIISTGVGQHQMWAAQYYKYKKPRQWLTSGG
LGAMGFGLPAAIGAAVGRPGEIVVDIDGDGSFIMNVQELATIKVENLPVKIMLLNNQHLGMVVQWEDRFYKANRAHTYLGDPS
NEEEIFPNMLKFAEACGVPAARVSHRDDLRAAIQKMLDTPGPYLLDVIVPHQEHVLPMIPSGGAFKDVITEGDGRCSY*

Figure S2. SlALS1 wild-type protein sequence

The SlALS1 Q184 and P186 are highlighted in purple.

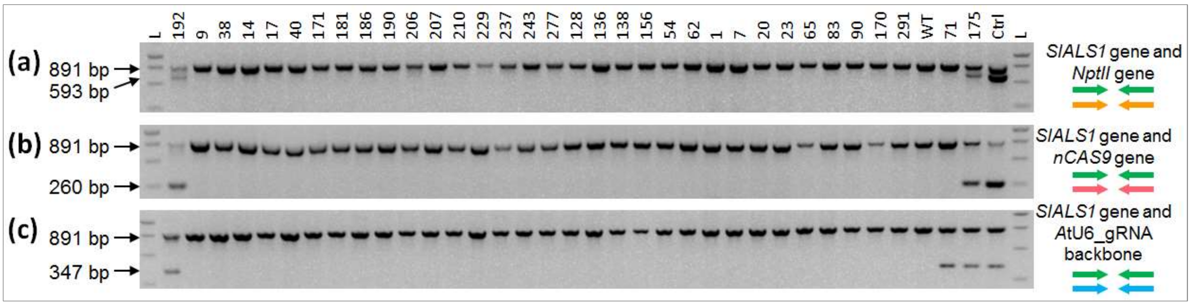

Figure S3. Molecular analysis of thirty plants without T-DNA insertion

Detection of the T-DNA was performed by multiplex PCR analysis on the thirty *SlALS1* edited plants without T-DNA (9, 38, 14, 17, 40, 171, 181, 186, 190, 206, 207, 210, 229, 237, 243, 277, 128, 136, 138, 156, 54, 62, 1, 7, 20, 23, 65, 83, 90, 291), the wild-type (WT), a plant carrying a potentially chimerical editing event (170), the PCR positive control (Ctrl), one *SlALS1* edited plant containing a fragment of T-DNA (71) and two *SlALS1* edited plant containing a T-DNA (192, 175). L: molecular marker. (**a**) Amplification of the *SlALS1* gene and the *NptII* gene. (**b**) Amplification of the *SlALS1* gene and the *Cas9* gene. (**c**) Amplification of the *SlALS1* gene and the *At*U6-gRNA backbone.

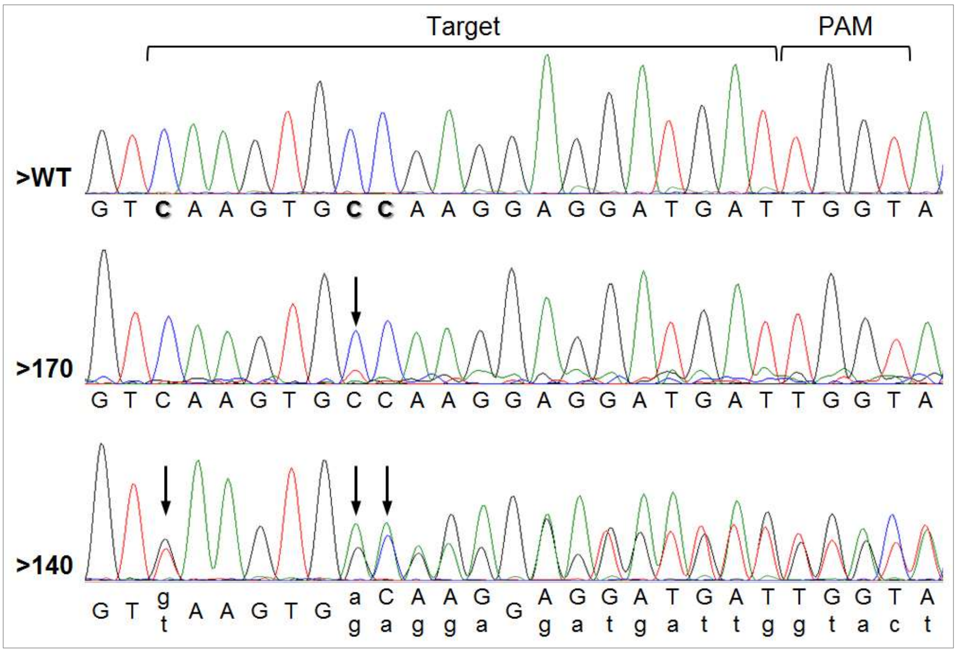

Figure S4. Sequencing chromatograms of the targeted *SlALS1* region of the wild-type (WT), a potential chimerical event (170) and an indel event (140).

The three targeting cytidines are bolded in the wild-type sequence. The two independent plantlets (170, 140) are chlorsulfuron resistant. The black arrows indicate potential modifications. Primers used for this sequencing are SlALS1.F and SlALS1.R.

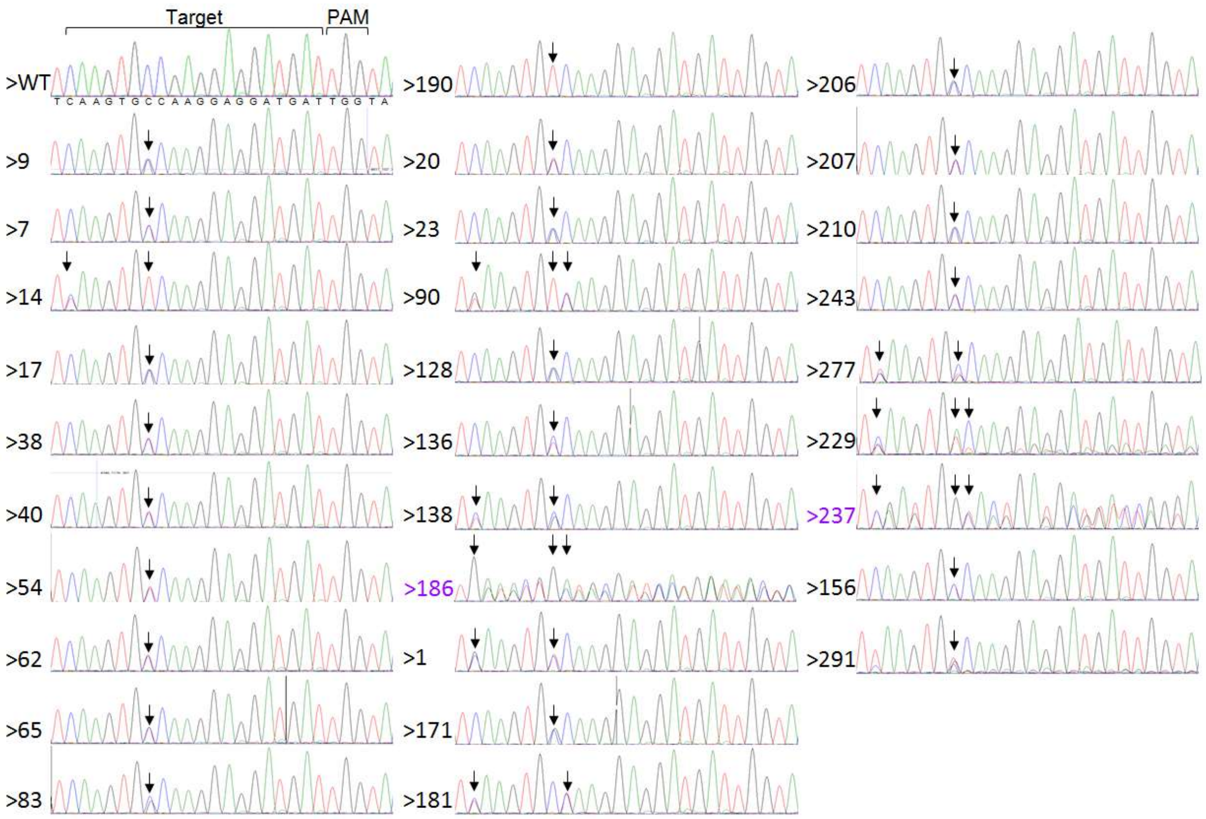

Figure S5. Sequencing chromatograms of the targeted *SlALS1* region of the wild-type (WT) and the thirty *SlALSI* edited plants without T-DNA insertion

The black arrows indicate modifications on C_{-20}, C_{-14} or C_{-13}. Purple writing indicates the presence of indels in the sequence. Primers used for this sequencing are SlALS1.F and SlALS1.R.

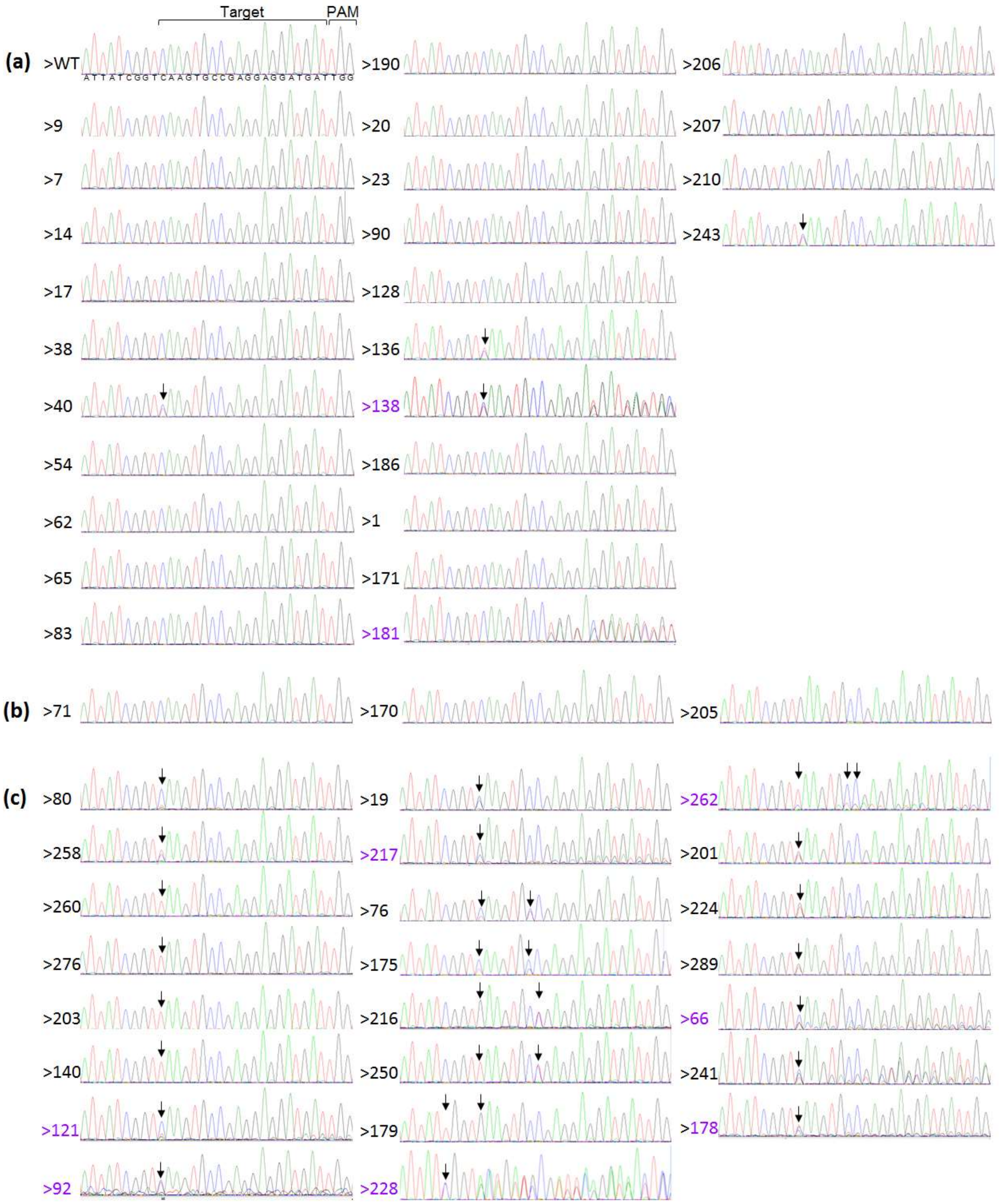

Figure S6. Sequencing chromatograms of the *SlALS2* locus of the wild-type (WT) and fifty one *SlALSI* edited plants

The black arrows indicate modifications on targeted cytidines. Purple writing indicates the presence of indels in the sequence. (**a**) Sequencing chromatograms of 25 *SlALS1* edited plants without T-DNA insertion. Four plantlets are edited on *SlALS2* locus (>40, >136, >138, >243). (**b**) Sequencing chromatograms of 3 *SlALS1* edited plants with T-DNA insertion, and without editing on *SlALS2* locus. (**c**) Sequencing chromatograms of 23 *SlALS1* edited plants with T-DNA insertion, and with editing on *SlALS2* locus. Primers used for this sequencing are SlALS2.F and SlALS2.R.

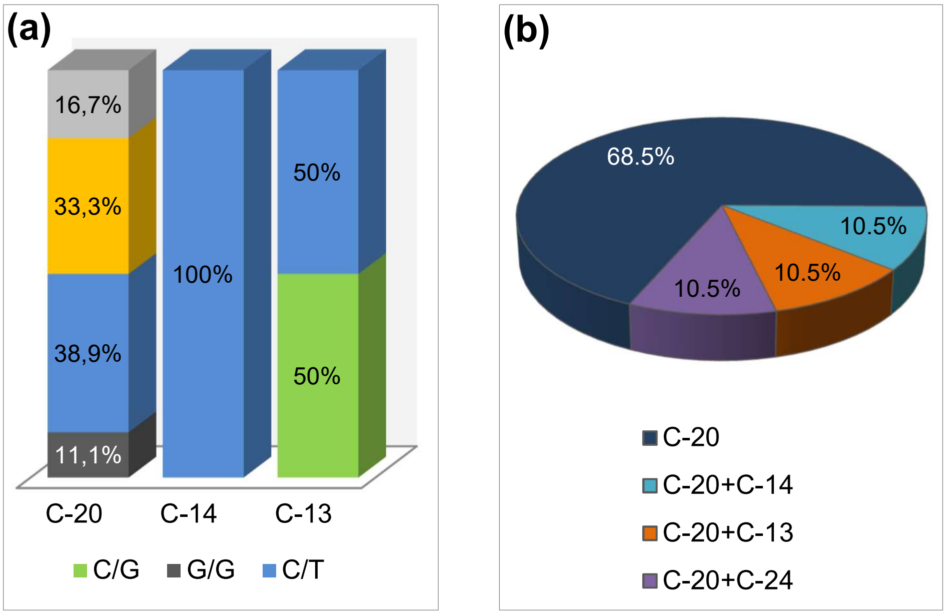

Figure S7. Base editing outcomes at the *StALS2* locus

(**a**) Percentage of each type of nucleotide changes found on cytidines C_{-20}, C_{-14} or C_{-13}. The total number of edited plants used in the study was 18 for C_{-20}, 2 for C_{-14}, 2 for C_{-13}. (**b**) Percentage of single editing event: C_{-20} in dark blue, double editing events: C_{-20}+C_{-14} in clear blue, and C_{-20}+C_{-13} in orange and C_{-20}+C_{-24} in purple. 19 edited plants were used in the study.

> *StALS1* gene sequence (*PGSC0003DMG400034102*)

ATGGCGGCTGCTGCCTCACCATCTCCATGTTTCTCCAAAACCCTACCTCCATCTTCCTCCAAATCTTCCACCATTCTTCCTAGATCTACCTTCC
CTTTCCACAATCACCCTCAAAAAGCCTCACCCCTTCATCTCACCCACACCCATCATCATCGTCGTGGTTTCGCCGTTTCCAATGTCGTCATATC
CACTACCACCCATAACGACGTTTCTGAACCTGAAACATTCGTTTCCCGTTTCGCCCCTGACGAACCCAGAAAGGGTTGTGATGTTCTTGTGGAG
GCACTTGAAAGGGAGGGGGTTACGGATGTATTTGCGTACCCAGGAGGTGCTTCTATGGAGATTCATCAGGCTTTGACACGTTCGAATATTATTC
GTAATGTGCTGCCACGTCATGAGCAAGGTGGTGTGTTTGCTGCAGAGGGTTACGCACGGGCGACTGGGTTCCCTGGTGTTTGCATTGCTACCTC
TGGTCCGGGAGCTACGAATCTTGTTAGTGGTCTTGCGGATGCTTTGTTGGATAGTATTCCGATTGTTGCTATTACGGGT**CAAGTGCCGAGGAGG**
ATGATTGGTACTGATGCGTTTCAGGAAACGCCTATTGTTGAGGTAACGAGATCTATTACGAAGCATAATTATCTTGTTATGGATGTAGAGGATA
TTCCTAGGGTTGTTCGTGAAGCGTTTTTTCTAGCGAAATCGGGACGGCCTGGGCCGGTTTTGATTGATGTACCTAAGGATATTCAGCAACAATT
GGTGATACCTAATTGGGATCAGCCAATGAGGTTGCCTGGTTACATGTCTAGGTTACCTAAATTGCCTAATGAGATGCTTTTGGAACAAATTATT
AGGCTGATTTCGGAGTCGAAGAAACCTGTTTTGTATGTGGGTGGTGGGTGTTTGCAATCAAGTGAGGAGCTGAGACGATTTGTGGAGCTTACGG
GTATTCCTGTGGCGAGTACTTTGATGGGTCTTGGAGCTTTTCCAACTGGGGATGAGCTTTCCCTTCAAATGTTGGGTATGCATGGGACTGTGTA
TGCTAATTATGCTGTGGATGGTAGTGATTTGTTGCTTGCATTTGGGGTGAGGTTTGATGATCGAGTTACTGGTAAATTGGAAGCTTTTGCTAGC
CGGGCGAAAATTGTCCATATTGATATTGATTCGGCTGAGATTGGAAAGAACAAGCAACCTCATGTTTCCATTTGTGCAGATATCAAGTTGGCAT
TACAGGGTTTGAATTCCATATTGGAGGGTAAAGAAGGTAAGCTGAAGTTGGACTTTTCTGCTTGGAGACAGGAGTTAACGGAACAGAAGGTGAA
GTACCCATTGAGTTTTAAGACTTTTGGTGAAGCCATCCCTCCACAATATGCTATTCAGGTTCTTGATGAGTTAACTAACGGAAATGCCATTATT
AGTACTGGTGTGGGGCAACACCAAATGTGGGCTGCCCAATACTATAAGTACAAAAAGCCACGCCAATGGTTGACATCTGGTGGATTAGGAGCAA
TGGGATTTGGTTTGCCTGCTGCAATAGGTGCGGCTGTTGGAAGACCGGGTGAGATTGTGGTTGACATTGATGGTGACGGGAGTTTTATCATGAA
TGTGCAGGAGTTAGCAACAATTAAGGTGGAGAATCTCCCAGTTAAGATTATGTTGCTGAATAATCAACACTTGGGAATGGTGGTTCAATGGGAG
GATCGATTCTATAAGGCTAACAGAGCACACACTTACTTGGGTGATCCTGCTAACGAGGAAGAGATCTTCCCTAATATGTTGAAATTCGCAGAGG
CTTGTGGCGTACCTGCTGCAAGAGTGTCACACAGGGATGATCTTAGAGCTGCCATTCAAAAGATGTTAGACACTCCTGGGCCATACTTGTTGGA
TGTGATTGTACCTCATCAGGAGCACGTTCTACCTATGATTCCCAGCGGCGGTGCTTTCAAAGATGTGATCACAGAGGGTGATGGGAGACGTTCC
TAT**TGA**

> *StALS2* gene sequence (*PGSC0003DMG400007078*)

ATGGCGGCTGCATCTCCATCTCCTTGTTTTTCCAAAAACCTACCTCCATCTTCATCAAAATCTTCCATCCTTCTTCCCAAATCTACCTTTACTT
TCCACAATCACCCCAAAAATACCTCACCCCTTCACCTTACCCACACCCAACATCATAGCCGTTTCACTGTCTCAAATGTCATCCTATCAACCAC
GACCCATAACGACGTTTCTGAACCCGAAATCTTCGTTTCACGTTTCGCCCCTGACGAACCCAGAAAGGGTTGTGATGTTCTTGTGGAGGCACTT
GAAAGGGAAGGGGTTAAGGATGTATTTGCATACCCAGGAGGTGCTTCCATGGAGATTCATCAGGCTTTGACACGTTCCAATATTATTCGTAATG
TGCTGCCACGTCATGAACAGGGTGGTGTGTTTGCTGCAGAGGGTTACGCACGGGCCACTGGGTTCCCTGGTGTTTGCATTGCTACATCTGGTCC
GGGAGCTACGAATCTTGTTAGCGGTCTTGCGGATGCTTTGTTGGATAGTATTCCGATTGTTGCTATTACGGGTCAAGTGCCGAGGAGGATGATT
GGTACTGATGCGTTTCAGGAAACTCCTATTGTTGAGGTAACGAGATCCATTACGAAGCATAATTATCTTGTTATGGATGTAGAGGATATTCCTA
GGGTTGTTCGTGAAGCGTTTTTTCTAGCGAAATCGGGACGGCCTGGACCGGTTCTGATTGATGTTCCTAAGGATATTCAGCAACAATTGGTGAT
ACCTAATTGGGATCAGCCAATGAGGTTGCCTGGTTACATCTCTAGGTTGCCTAAATTGCCTAATGAGATGCTTTTGGAACAAATTGTTAGGCTG
ATTTCGGAGTCGAAGAAGCCTGTTTTGTATGTGGGTGGTGGGTGTACACAATCGAGTGAGGAGCTGAGACGATTTGTGGAGCTTACGGGTATTC
CTGTGGCGAGTACTTTGATGGGTCTTGGAACTTTTCCATGTGGGGATGAGCTTTCTCTTCAAATGTTGGGTATGCATGGGACTGTGTATGCTAA
TTATGCGGTGGATAGTAGTGATTTGTTGCTTGCATTTGGGGTGAGGTTTGATGATCGAGTTACTGGTAAATTGGAAGCTTTTGCTAGCCGAGCG
AAAATTGTCCACATTGATATTGATTCGGCTGAGATTGGAAAGAACAAGCAACCTCATGTTTCCATTTGTGCAGATATCAAGTTGGCATTACAGG
GTTTGAATTCCATATTGGAGGGTAAAGAAGGTAAGCTGAAGTTGGACTTTTCTGCTTGGAGGCAGGAGTTAACGGAGCAGAAGGTGAAGTACCC
ATTGAATTATAAGACTTTTGGTGAAGCCATCCCTCCACAATATGCTATTCAGGTTCTTGATGAGTTAACTAACGGAAATGCCATTATTAGTACT
GGTGTGGGGCAACACCAAATGTGGGCTGCCCAATACTATAAGTACAAAAAGCCACGCCAATGGTTGACATCTGGTGGATTAGGAGCAATGGGAT
TTGGTTTGCCTGCTGCTATAGGTGCGGCTGTTGGAAGACCGGGTGAGATTGTGGTTGACATTGATGGTGACGGGAGTTTTATCATGAATGTGCA
GGAGTTAGCAACAATTAAGGTGGAGAATCTCCCAGTTAAGATTATGTTACTGAATAATCAACACTTGGGAATGGTGGTTCAGTGGGAGGATCGA
TTCTATAAGGCTAACAGAGCACACACTTACTTGGGTAATCCTGCTAATGAGGAAGAAATCTTCCCTAATATGCTGAAATTTGCAGAGGCTTGTG
GCGTACCTGCTGCAAGAGTGTCACACAGGGATGATCTTAGAGCTGCCATTCAAAAGATGTTAGACACTCCTGGGCCATACTTGTTGGATGTGAT
TGTACCTCATCAGGAGCACGTTCTACCTATGATTCCCAGTGGTGGCGCTTTCAAAGATGTGATTACGGAGGGTGATGGGAGACGTTCCTAT**TGA**

>StALS1 protein sequence (XP_006361740)

MAAAASPSPCFSKTLPPSSSKSSTILPRSTFPFHNHPQKASPLHLTHTHHHRRGFAVSNVVISTTTHNDVSEPETFVSRFAPDEPRKGCDVLVE
ALEREGVTDVFAYPGGASMEIHQALTRSNIIRNVLPRHEQGGVFAAEGYARATGFPGVCIATSGPGATNLVSGLADALLDSIPIVAITGQVPRR
MIGTDAFQETPIVEVTRSITKHNYLVMDVEDIPRVVREAFFLAKSGRPGPVLIDVPKDIQQQLVIPNWDQPMRLPGYMSRLPKLPNEMLLEQII
RLISESKKPVLYVGGGCLQSSEELRRFVELTGIPVASTLMGLGAFPTGDELSLQMLGMHGTVYANYAVDGSDLLLAFGVRFDDRVTGKLEAFAS
RAKIVHIDIDSAEIGKNKQPHVSICADIKLALQGLNSILEGKEGKLKLDFSAWRQELTEQKVKYPLSFKTFGEAIPPQYAIQVLDELTNGNAII
STGVGQHQMWAAQYYKYKKPRQWLTSGGLGAMGFGLPAAIGAAVGRPGEIVVDIDGDGSFIMNVQELATIKVENLPVKIMLLNNQHLGMVVQWE
DRFYKANRAHTYLGDPANEEEIFPNMLKFAEACGVPAARVSHRDDLRAAIQKMLDTPGPYLLDVIVPHQEHVLPMIPSGGAFKDVITEGDGRRS
Y

>StALS2 protein sequence (XP_006348357)

MAAASPSPCFSKNLPPSSSKSSILLPKSTFTFHNHPKNTSPLHLTHTQHHSRFTVSNVILSTTTHNDVSEPEIFVSRFAPDEPRKGCDVLVEAL
EREGVKDVFAYPGGASMEIHQALTRSNIIRNVLPRHEQGGVFAAEGYARATGFPGVCIATSGPGATNLVSGLADALLDSIPIVAITGQVPRRMI
GTDAFQETPIVEVTRSITKHNYLVMDVEDIPRVVREAFFLAKSGRPGPVLIDVPKDIQQQLVIPNWDQPMRLPGYISRLPKLPNEMLLEQIVRL
ISESKKPVLYVGGGCTQSSEELRRFVELTGIPVASTLMGLGTFPCGDELSLQMLGMHGTVYANYAVDSSDLLLAFGVRFDDRVTGKLEAFASRA
KIVHIDIDSAEIGKNKQPHVSICADIKLALQGLNSILEGKEGKLKLDFSAWRQELTEQKVKYPLNYKTFGEAIPPQYAIQVLDELTNGNAIIST
GVGQHQMWAAQYYKYKKPRQWLTSGGLGAMGFGLPAAIGAAVGRPGEIVVDIDGDGSFIMNVQELATIKVENLPVKIMLLNNQHLGMVVQWEDR
FYKANRAHTYLGNPANEEEIFPNMLKFAEACGVPAARVSHRDDLRAAIQKMLDTPGPYLLDVIVPHQEHVLPMIPSGGAFKDVITEGDGRRSY

Figure S8: *Acetolactate synthase StALS1-2* gene and StALS1-2 protein sequences

The start codon is represented with red letters and the stop codon is represented with bold letters. The target sequence is highlighted in blue. The three targeting cytidines are highlighted in red. The PAM site is highlighted in green. The StALS1 Q184 and P186 are highlighted in purple.

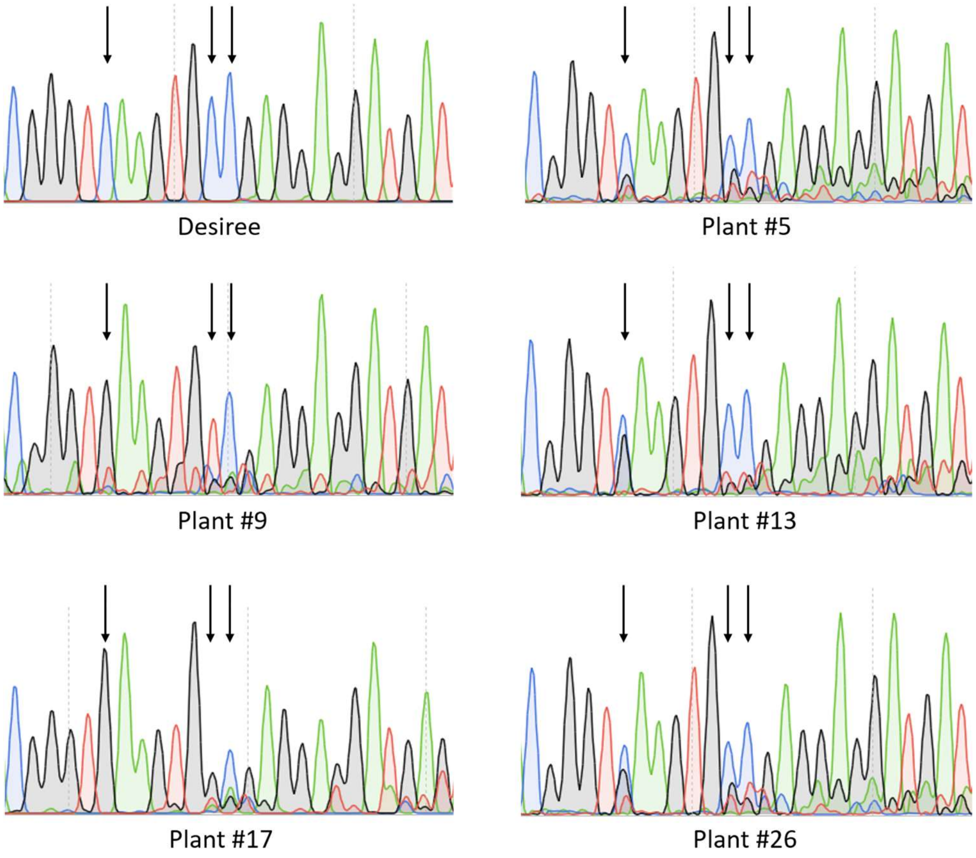

Figure S9: Sequencing chromatograms of the targeted region (*StALS1* and *StALS2*) of Desiree and some mutants harbouring indels.

The black arrows indicate the targeted cytidines.

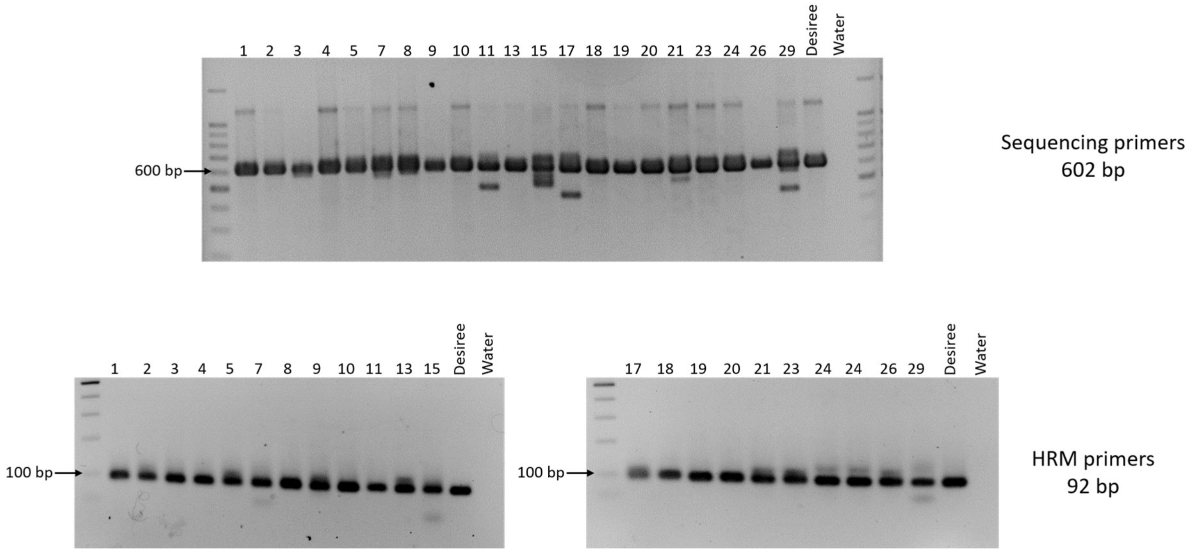

Figure S10: PCR genotyping of the targeted region (*StALS1* and *StALS2*). PCR products were run on an agarose gel for the detection of band-shifts, indicating the presence/absence of indels in the targeted region.

Table S1

List of primers used in tomato

**Target**	**Primer ID**	**Sequence (5'-3')**	**Size (bp)**
PCR detection/sequencing of the *SlALS1* gene	→	GGCACTTGAAAGGGAAGG	891
	←	CCAATCTCTGCCGAATCAAT	
PCR detection of the *nCAS9* gene	→	TCCCTTACTACGTGGGACCTC	260
	←	CATTCCCTCGGTCACGTACT	
PCR detection of the *NptII* gene	→	AGACAATCGGCTGCTCTGAT	593
	←	AGCCAACGCTATGTCCTGAT	
PCR detection of the *At*U6-gRNA backbone	→	TCCCAGGATTAGAATGATTAGG	347
	←	CGGTGCCACTTTTTCAAGTT	
HRM detection of the *SlALS1* gene	→	CTTGTTAGTGGTCTTGCGGATG	104
	←	GTTTCCTGGAACGCATCAGTACC	
Sequencing of the *SlALS1* gene	SlALS1.F	GTGGTTTTGCCGTTGCCAAT	761
	SlALS1.R	CAGCTCCTCACTTGATTGCG	
Sequencing of the *SlALS2* gene	SlALS2.F	GCCGTTTCACTGTTTCAAAT	761
	SlALS2.R	CAGCTCCTCACTCGACTGTG	

Table S2

List of primers used in potato

**Target**	**Primer ID**	**Sequence (5’-3’)**	**Size (bp)**
Sequencing of the *StALS1* and *StALS2* genes	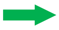	GATGTTCTTGTGGAGGCACTTG	602
	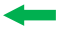	TTCTTCGACTCCGAAATCAGC	
PCR detection of the n*CAS9* gene	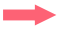	ACTTTTCGTTGAGCAGCACAA	350
	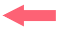	GATCAGCCCTTGAATCACCA	
PCR detection of the *NptII* gene	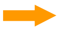	GATGGATTGCACGCAGGTTC	406
	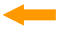	GATGTTTCGCTTGGTGGTCG	
PCR detection of the *At*U6-gRNA backbone	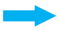	AAACATCATCCTCCTCGGCACTTG	207
	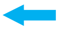	GATAATCTTCAAAAGGCCCCTGG	
PCR detection of the control gene (*StGBSSI*)		AGACCACACATCACATGGCAA	352
		CTTACTGCAAGGGCTGGTGG	
HRM of the *StALS1* and *StALS2* genes	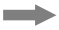	CTTGCGGATGCTTTGTTGG	92
	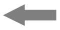	GTTTCCTGAAACGCATCAGTACC	
